# ITPI: Initial Transcription Process-Based Identification Method of Bioactive Components in Traditional Chinese Medicine Formula

**DOI:** 10.1155/2016/8250323

**Published:** 2016-02-29

**Authors:** Baixia Zhang, Yanwen Li, Yanling Zhang, Zhiyong Li, Tian Bi, Yusu He, Kuokui Song, Yun Wang

**Affiliations:** ^1^School of Chinese Pharmacy, Beijing University of Chinese Medicine, Beijing 100102, China; ^2^Institute of Information on Traditional Chinese Medicine of China, Academy of Chinese Medical Sciences, Beijing 100700, China; ^3^China Minority Traditional Medical Center, Minzu University of China, Beijing 100081, China

## Abstract

Identification of bioactive components is an important area of research in traditional Chinese medicine (TCM) formula. The reported identification methods only consider the interaction between the components and the target proteins, which is not sufficient to explain the influence of TCM on the gene expression. Here, we propose the Initial Transcription Process-based Identification (ITPI) method for the discovery of bioactive components that influence transcription factors (TFs). In this method, genome-wide chip detection technology was used to identify differentially expressed genes (DEGs). The TFs of DEGs were derived from GeneCards. The components influencing the TFs were derived from STITCH. The bioactive components in the formula were identified by evaluating the molecular similarity between the components in formula and the components that influence the TF of DEGs. Using the formula of Tian-Zhu-San (TZS) as an example, the reliability and limitation of ITPI were examined and 16 bioactive components that influence TFs were identified.

## 1. Introduction

Identification of the bioactive components present in a traditional Chinese medicine (TCM) formula using computational method not only is fast and efficient, but also has the prospect of explaining the mechanism of action. The main identification methods reported to date are computer virtual screening technology [1], network pharmacology [2], directed TCM grammar systems (dTGS) [3], system pharmacology [4], and so forth. These methods mainly consider the effect of the components that are present in a TCM formula on the expressed proteins and do not sufficiently explain why a TCM formula affects the expression of a number of proteins. Here, we propose a new method, the Initial Transcription Process-based Identification (ITPI) method, to characterize the bioactive components present in a TCM formula. This method identifies the bioactive components by considering the potential interaction between the components and the transcription factors (TFs) of differentially expressed genes (DEGs). When used in combination with the previously reported methods, the ITPI method can not only help explain the mechanism of action, but also enable a more comprehensive discovery of the bioactive components that are present in a formula.

## 2. Methods and Materials

### 2.1. Principle of ITPI

**Figure 1:**
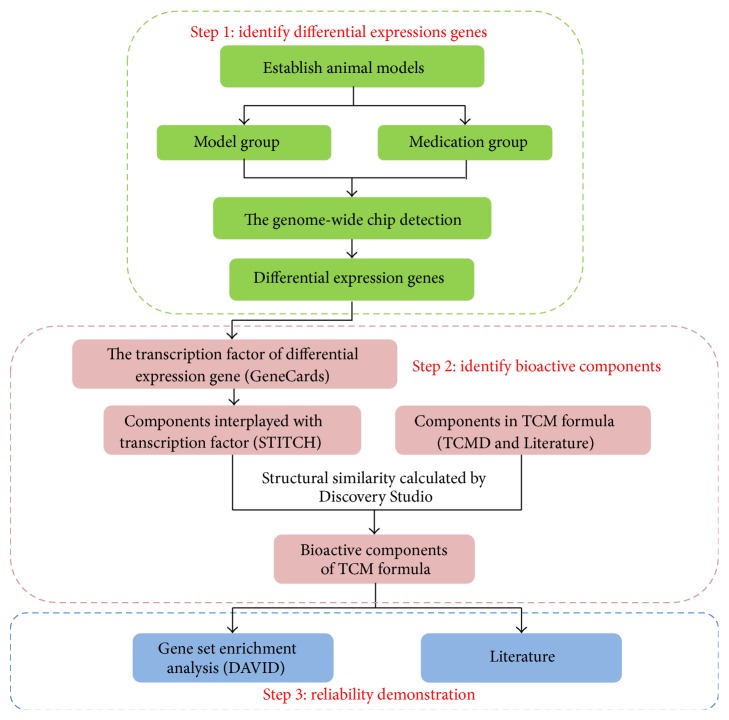
The workflow of ITPI.

Previous studies have shown that TCM formulas can influence gene expression [5, 6]. However, the reported methods only consider the influence of the TCM on expressed proteins and fail to explain the role of the TCM on gene expression. Transcription is an important event during gene expression. TFs are key players in the transcription and are also drug targets [7, 8], but they are usually ignored. TFs may be the targets of a given TCM formula, and the interactions of the formula with TFs may be an important mechanism by which the TCM formula exerts its biological effect. Therefore, the present study established the ITPI method to characterize the bioactive components present in a TCM formula. Here, we also discuss each step of the ITPI method (Figure 1) in detail.

The expression level of each gene in the medication and model groups is detected by the genome-wide chip detection technology. The normalized data are expressed as means ± standard deviation (SD). Comparisons between the medication and model groups are then performed by one-way analysis of variance (ANOVA), followed by Student's *t*-test. Values of *P* < 0.05 is considered statistically significant, and genes with significant expression levels are regarded to be DEGs.

With the development of bioinformatics and chemoinformatics, many TF databases [9, 10] and the component-protein interaction databases [11] have been established. Most of these databases contain the data on various organisms, including human, rat, and mouse. Therefore, care should be taken in choosing an organism with regard to the specific purpose of the investigation. In the ITPI method, the most relevant TFs of DEGs are derived from GeneCards (http://www.genecards.org/), and the potential components that can interact with the TF are derived from the STITCH (http://stitch.embl.de/). In STITCH, the potential components that can interact with TFs are identified by name or SMILES string. The required confidence is higher than 0.400, and the active prediction methods include gene fusion, cooccurrence, coexpression, experiments, databases, neighborhood, predictions, and text mining.

To date, many studies on the components of TCM formulas have been carried out and a large number of components have been separated and identified. The known components of a given TCM formula can be derived from the literature, which is included in China National Knowledge Internet (CNKI), PubMed (1979~2015), and the traditional Chinese medicines database (TCMD) [12]. The name, structure, and the SMILES of the known components should be recorded. Finally, repetition of the name of the components, both in the literature and in TCMD, is eliminated. When the component exists as a synonym, the repeated component name is identified by structure, and these instances are eliminated.

Molecular similarity is an important theme in chemoinformatics [13]. Its use is based on the principle that compounds with similar structures exhibit similar chemical properties and biological activities [14]. Although molecular similarity can also be calculated using molecular properties, such as log⁡*P* and molecular weight, it is mostly defined as a distance measure in a structural or physicochemical descriptor space. There are many different distance measures available. These measures include Tanimoto, Dice, Cosine, and Soergel [15]. Concerning the fingerprint properties, the most popular similarity measure used for comparing the chemical structures represented by fingerprints is the Tanimoto (or Jaccard) coefficient *T* [15]. Therefore, the Tanimoto coefficient was selected for the determination of molecular similarity. Two structures are usually considered to be similar if *T* > 0.85. After the similarity calculation, the components that potentially interact with TFs can be determined. The Tanimoto coefficient is defined as follows:

(1) $$\begin{array}{c} \text{Tanimoto coefficient}=\frac{SA}{\left(SA+SB+SC\right)}, \end{array}$$

where SA is the number of AND bits (bits present in both the target component and the reference component); SB is the number of bits present in the target component, but not in the reference component; and SC is the number of bits present in the reference component, but not in the target component.

Using this method, the bioactive components that are present in the TCM formula that can interact with TFs can be identified, indicating which bioactive components influence the expression of DEGs. The reported identification methods consider the interaction between the components and proteins expressed by DEGs, whereas ITPI identifies the bioactive components from the initial transcription process and regards TFs as drug targets. Combining other methods with ITPI, the bioactive components can be comprehensively discovered along the overall regulation process of TCM formula.

### 2.2. Application of ITPI on TZS

Tian-Zhu-San (TZS), which consists of two Chinese medicinal herbs, Gastrodiae Rhizoma and Trillium, is mainly used for the treatment of neurodegenerative diseases in the Tujia ethnic minority [16]. Gastrodiae Rhizoma is the tuber of* Gastrodia elata* BL. (Orchidaceae) and Trillium is the rhizome of* Trillium tschonoskii* Maxim. (Liliaceae). Although many studies have attempted to identify the bioactive components that are present in TZS [17–20], the component that acts against neurodegenerative diseases remains unknown. TZS can influence gene expression and likely interacts with TFs.

#### 2.2.1. Preparation of TZS Superfines

Gastrodiae Rhizoma and Trillium were purchased from the Anguo (Hebei Province). Dr. Yanwen Li of the Institute of Information on Traditional Chinese Medicine of China performed the identification and authentication of the samples. The dried herbs were ground into superfine powder (grain diameter ≤ 15 *μ*m) using an Ultra-Micro Pulverizer. The superfine powder was dissolved in distilled water and stored at 4°C before its administration to rats.

#### 2.2.2. Animals

Male Sprague-Dawley rats weighing within 190~219 g (Beijing Vital River Laboratory Animal Technology Co. Ltd.) were used for* in vivo* experiments. The animals were housed at 21 ± 2°C under a 12-hour light/12-hour dark cycle (lights on at 07:00), were allowed to acclimatize, and were provided free access to water and food for extra week. All animal care and experimental procedures were performed in accordance with the National Institutes of Health Guide for the Care and Use of Laboratory Animals. The procedures were approved by the Committee on Animal Care of the Beijing University of Chinese Medicine. All efforts were made to minimize animals suffering and to reduce the number of animals used in the experiments.

#### 2.2.3. Model Preparation of Vascular Dementia Induced by Chronic Cerebral Hypoperfusion and Drug Delivery

All procedures were performed according to the reported methods [21]. The animals were injected with 10% chloral hydrate (0.35 g·Kg^−1^) intraperitoneally. After administration of anesthesia, the animals were placed in a supine position and a median incision of approximately 1.5 to 2 cm was made on the neck. The bilateral common carotid arteries were isolated and ligated, and the incision was sutured. The animals were fed with the same diet for 60 days under the same conditions (Statement: our laboratory has established a stable method to duplicate the model of vascular dementia induced by chronic cerebral hypoperfusion. Based on the research purposes, this study omitted the neurobehavioral evaluation section).

The surviving rats were randomly divided into model and medication groups. After 24 hours, 625 mg·Kg^−1^ superfine powder of TZS (the effective dose was calculated by the method of body weight) was administered to the medication group, and the blank and model groups were administered distilled water. This intragastric administration was continued for 60 days in all groups.

#### 2.2.4. The Genome-Wide Chip Detection

The hippocampal tissues of the medication and model group rats were taken and stored in liquid nitrogen. Total RNA was extracted by the Trizol method and then purificated. The RNA quality of RNA was assessed by agarose gel electrophoresis. Then, using the purified total RNA, double stranded cDNA was synthesized, which was then fragmented and fluorescently labeled.

Fragmented cDNA and the reference reagents were then mixed, and the hybridization solution was prepared. The hybridization solution was poured onto an Affymetrix GeneChip Rat Gene 2.0ST Array. After 16 hours of hybridization, the chip was removed from the Hybridization Oven 645. With the adding of eluent and staining fluid, the wash and staining were completed in fluidics station 450.

The microarray results were scanned using a GeneChip Scanner 3000, and the original data were read using the Command ConsoleSoftware 3.1. After normalizing the data, the signal strength of the medication group and model groups was compared. If the ratio was >2, the gene was considered to be the significantly upregulated expression gene, and if the ratio was <0.5, the gene was considered to be significantly downregulated expression gene.

#### 2.2.5. Discover the Bioactive Components of TZS for Antivascular Dementia

Analyses of the gene expression data, the TFs of the DEGs, the components that can interact with the TFs of the DEGs, the components of the TCM formula, and the molecular similarity were all carried out according to the principles of ITPI. It should be noted that when components existed as synonyms, we deleted the repeated component names, using the “ChemBioFinder for Office 12.0.” Molecular similarity was calculated using the “Find Similar Molecules by Fingerprints” module of the Discovery Studio 4.0 [22].

### 2.3. Validation of Identification Results

To demonstrate the rationality of ITPI, we performed the following: verifying the relationship between the pharmacological action of the bioactive components and vascular dementia by looking up literature and the other was verifying whether the DEGs which were regulated by bioactive components related to vascular dementia by gene set enrichment analysis [23]. Gene-GO term enrichment analysis was performed to highlight the most relevant biological pathways associated with a given gene list. DAVID 6.7 Functional Annotation Clustering was used for this purpose. *P* < 0.05 indicated significantly enriched biological pathways.

## 3. Results and Discussions

### 3.1. Bioactive Components of TZS for Antivascular Dementia

After normalizing the results from genome-wide chip detection, we compared the medication group and model groups (Supplemental Information 1 in Supplementary Material available online at http://dx.doi.org/10.1155/2016/8250323) and found 229 DEGs, which included 219 upregulated genes (>2) and 10 downregulated genes (<0.5) (Supplemental Information 2). The TFs of the DEGs (Supplemental Information 3) and the components that interacted with the TFs (Supplemental Information 4) are listed in the Supplementary Material. A total of 52 components (Supplemental Information 5) of TZS were used to calculate the molecular similarity. The results of the molecular similarity calculations revealed that there are 16 components whose Tanimoto coefficients were greater than 0.85 and involved 13 TFs and 30 DEGs (Table 1).

These results showed that one component can regulate several TFs or genes; different components can regulate the same gene on account of their interaction with the same TF; that is to say, one gene or TF can be affected by multiple components. This indicates that multiple bioactive components of TZS interplay with multiple genes or TFs and produce the antivascular dementia effect.

### 3.2. Validation of Identification Results

30 DEGs were used for the enrichment analysis, and 10 DEGs were significantly enriched in 7 clusters (Table 2). The pathogenesis of vascular dementia mainly involves the cholinergic system, inflammatory processes, oxygen free radicals, and the transport of NO, among others [24–26]. These enriched biopathways, such as blood circulation [27], oxidation reduction [28], hormone metabolic process [29], and oxygen transport [30], were all found to be closely related to vascular dementia. That is to say, the DEGs of* Hba-a2*,* F5*,* Nts*,* Cyp11b3*,* Cyp2j4*,* Cyp2a2*,* Nqo2*,* Ttr*,* Ngf*, and* Hbb* were also found to be closely related to vascular dementia.

The results of enrichment analysis showed that 10 of the 30 DEGs were closely related to vascular dementia. These 10 DEGs were found to be regulated by 16 components. This indicated that 16 components were indeed effective against vascular dementia and that TZS acts against antivascular dementia mainly by regulating these 10 DEGs (Table 1). Gene set enrichment analysis that is based on the DEGs is a commonly used method. The enrichment analysis, which was based on the 30 DEGs regulated by the bioactive components, was more focused and specific.

A literature search can be used to identify some of the vascular dementia-related pharmacological effects of the components. For example, it has reported that bis(4-hydroxybenzyl)ether exerts a neuroprotective effect in an ischemic model [31], *β*-sitosterol protects neural stem cells from neurodegenerative diseases [32], diosgenin has an anti-inflammatory effect and acts against oxidative during the monocrotaline-induced pulmonary hypertension in rats [33, 34], and daucosterol induces a protective Th1 immune response against disseminated Candidiasis in mice [35]. Although there is no direct proof of the interaction between the components and the respective TFs or genes, the studies described above are indicative of the rationality of using ITPI for the identification of bioactive components to some extent.

## 4. Conclusion

Using the newly developed ITPI method, we identified 16 components of TZS and 10 DEGs that are closely related to vascular dementia. The results demonstrated the utilizing of ITPI in the identification of bioactive components and its rationality. The ITPI method focuses on the overall components of a given TCM formula and genes of treatment objects, along with the initial transcription process of “all of the components of TCM formula-TFs-DEGs.” Compared with the reported methods, such as computer virtual screening technology and network pharmacology, ITPI could not only identify the bioactive components but also help explain why a given TCM formula influences the gene expression. The ITPI method also identifies the targeted TFs and genes of the bioactive components by combining the methods of technology of bioinformatics and cheminformatics. Meanwhile, the results suggest that a TCM formula exerts its therapeutic effect by regulating multiple TFs through the action of multiple components.

The ITPI method still has some limitations. Gene expression is controlled at multiple levels, including transcription, and TFs can regulate the initial process of transcription. Interaction with TFs may be only one of the many ways in which TCM exerts its biological effect, but its small impact could produce a huge change just because initial transcription process was the upstream of the biological pathway. Because the results were based on existing data included in databases, the genes in the similarity calculation only accounted for a portion of the total amount of DEGs; the data of TFs regulated DEGs remains to be perfect. Further studies on the TCM formula components, and the related TFs and genes, along with improved databases, will enable a more comprehensive analysis and detailed characterization of the bioactive components. The correlation coefficient and the action mode of the interplay between the components and the TFs were not considered in the present study. In addition, the way that compounds affect TFs is indirect or direct but unclear. Therefore, we could not describe the potency and the action mode of the TZS components that interplay with TFs and DEGs.

ITPI identifies bioactive components based on the TFs that they influence. The results will be more comprehensive if ITPI is used in tandem with other methods. In this study, we discovered that TZS cured vascular dementia by affecting blood circulation and oxygen transport and by regulating hormone levels. Our further goal is to draw a detailed biological network, describe the relationship between these bioactive components and biomolecules, and explain the mechanism by which the components of TZS act against vascular dementia at a molecular level. We can also carry out the drug repositioning by reversing the ITPI, screen the components that target specific genes or TFs associated with a disease, and find a specific drug combination for treating a given disease. The present study provides a novel platform for the identification of bioactive components present in a TCM formula, which can be applied more widely in the research of TCM formula studies.

## Supplementary Material

Supplemental Information 1 shows the expression of all genes in black, model and mediated group. The results of mediated vs model also included. Supplemental Information 2 shows the 219 upregulated genes and 10 downregulated genes. Supplemental Information 3 shows the TFs of the DEGs. Supplemental Information 4 shows the components that interacted with the TFs. Supplemental Information 5 shows the 52 components of TZS.

## Figures and Tables

**Table 1:** Sixteen bioactive components and their regulated TFs and DEGs.

TCM	TZS components	The CID of component that interacts with TF	Tanimoto coefficient	TF	Differential expression genes
*Gastrodia elata* BL.	4,4′-Dihydroxydiphenyl methane	6623	0.877023	ER-alpha	*F5*/*Hba-a2*/*Rnase111*
	*β*-Sitosterol	2371	0.915282	Sp1	*Rnase4*/*F5*/*Slc19a3*
		2524	0.896709	Egr-1	*Ifitm7*
	Citric acid	311	1	Olf-1	*Cyp2a2*/*Cyp2j4*
	3-Hydroxy-3-methoxycarbonylpentanedioic acid	311	0.8	Olf-1	*Cyp2a2*/*Cyp2j4*
	Daucosterol	2371	0.879428	Sp1	*Rnase4*/*F5*/*Slc19a3*
		2524	0.852015	Egr-1	*Ifitm7*
	Margaric acid	985	0.994764	Sp1	*Rnase4*/*F5*/*Slc19a3*
		2506	0.941581	Sp1	*Rnase4*/*F5*/*Slc19a3*
		148177	0.875424	Sp1	*Rnase4*/*F5*/*Slc19a3*
	Bis(4-Hydroxybenzyl)ether	6623	0.850932	ER-alpha	*F5*/*Hba-a2*/*Rnasel11*
	Vitamin A	5538	0.970093	c-Fos	*Dusp12*/*Gpatch4*/*Ngf*/*Nts*/*Hba-a2*/*RGD1564469*
		1744	0.856672	CHOP-10	*Ttr*/*Kcnj13*
	Palmitic acid	985	1	Sp1	*Rnase4*/*F5*/*Slc19a3*
		2506	0.933009	Sp1	*Rnase4*/*F5*/*Slc19a3*
	Palmitoyl glycerol	2506	0.96445	Sp1	*Rnase4*/*F5*/*Slc19a3*
		985	0.94697	Sp1	*Rnase4*/*F5*/*Slc19a3*
		3987	0.916115	Egr-1	*Ifitm7*
		148177	0.884291	Sp1	*Rnase4*/*F5*/*Slc19a3*
		2499	0.852916	STAT5B	*Clic6*

*Trillium tschonoskii* Maxim.	Pennogenin	2524	0.890232	Egr-1	*Ifitm7*
		2371	0.882143	Sp1	*Rnase4*/*F5*/*Slc19a3*
		54454	0.863784	ATF-2	*Cyp2j4*/*Rasl11a*/*Slc16a13*
	Linoleic acid	1424	0.932432	RelA	*Cyp2j4*/*RT1-T24-4*/*Nqo2*
		3987	0.865169	Egr-1	*Ifitm7*
		1444	0.860317	STAT3	*Clic6*/*Hbb*/*Krt18*/*Fpr-rs3*/*Ifitm7*/*RGD1564126*/*Zfp467*
	4-Hydroxybenzoic acid	44540357	0.8679	c-Jun	*Dusp12*/*Gpatch4*/*Ngf*/*Fpr-rs3*/*Hba-a2*/*RGD1564469*/*S100vp*
	3-O-*α*-L-Rhamnopyranosyl (1→3) sterylglucoside	2371	0.879428	Sp1	*Rnase4*/*F5*/*Slc19a3*
		2524	0.852015	Egr-1	*Ifitm7*
	26-Chloro-26-deoxycryptogenin	2371	0.886708	Sp1	*Rnase4*/*F5*/*Slc19a3*
		2524	0.878365	Egr-1	*Ifitm7*
		146898	0.862239	STAT2	*Clic6*/*Naip5*/*Cyp11b3*
		54454	0.855305	ATF-2	*Cyp2j4*/*Rasl11a*/*Slc16a13*
		400769	0.853801	STAT3	*Clic6*/*Hbb*/*Krt18*/*Fpr-rs3*/*Ifitm7*/*RGD1564126*/*Zfp467*
		5753	0.853659	FOXO3	*Fpr-rs3*/*RGD1559708*
	Diosgenin	2371	0.885714	Sp1	*Rnase4*/*F5*/*Slc19a3*
		2524	0.881	Egr-1	*Ifitm7*
		54454	0.866091	ATF-2	*Cyp2j4*/*Rasl11a*/*Slc16a13*

The Tanimoto coefficient of these components was greater than 0.85; this table shows the corresponding relationship between the bioactive components, TFs and DEGs.

**Table 2:** The significantly enriched biopathways.

GO ID	Term	Count	*P* value	Genes
GO:0003013	Circulatory system process	4	0.001231	*Hba-a2*/*F5*/*Nts*/*Cyp11b3*
GO:0008015	Blood circulation	4	0.001231	*Hba-a2*/*F5*/*Nts*/*Cyp11b3*
GO:0055114	Oxidation reduction	5	0.009123	*Cyp2j4*/*F5*/*Cyp11b3*/*Cyp2a2*/*Nqo2*
GO:0042445	Hormone metabolic process	3	0.010025	*Ttr*/*Cyp11b3*/*Ngf*
GO:0015671	Oxygen transport	2	0.016748	*Hba-a2*/*Hbb*
GO:0010817	Regulation of hormone levels	3	0.020049	*Ttr*/*Cyp11b3*/*Ngf*
GO:0015669	Gas transport	2	0.025023	*Hba-a2*/*Hbb*

Values of* P *< 0.05 were considered as significantly enriched biopathways.
